# Evaluation of the Antimicrobial Activity of Phytochemicals from Tea and Agarwood Leaf Extracts against Isolated Bacteria from Poultry and Curd

**DOI:** 10.1155/2023/6674891

**Published:** 2023-11-07

**Authors:** Shah Rucksana Akhter Urme, Syeda Fahmida Ahmed, Mohammed Mostafa Al Quadir, Mst Rubaiat Nazneen Akhand, Mohammad Mehedi Hasan Khan

**Affiliations:** ^1^Department of Biochemistry & Chemistry, Sylhet Agricultural University, Sylhet 3100, Bangladesh; ^2^Department of Animal and Fish Biotechnology, Sylhet Agricultural University, Sylhet 3100, Bangladesh; ^3^Faculty of Biotechnology and Genetic Engineering, Sylhet Agricultural University, Sylhet 3100, Bangladesh

## Abstract

Antibiotic-resistant bacteria are becoming increasingly common, leading to a global health crisis. The effects of abusing antibiotics not only increase pathogenic resistance but also cause various diseases and syndromes. Gut microbiota contains many beneficial roles for health, while antibiotics kill both pathogens and gut microbiota which is considered one of the major side effects of antibiotics. In fact, new antibiotic compounds are needed in this urgent scenario; phytoremediation is the oldest but most effective method, and research on the antibacterial properties of several types of medicinal plants has already been conducted. Tea and agarwood plants are well known for their economic contribution in both beverage and cosmetic production, as well as for their medicinal value. In this study, tea and agarwood leaf extracts were analyzed for their antimicrobial activity against both pathogenic and beneficial bacteria. Fresh tea (*Camellia sinensis*) leaves were collected in three varieties, namely, BT-6 from Sylhet, BT-7 from Moulvibazar, and BT-8 from Habiganj; also, green tea (nonfermented tea), black tea (fully fermented tea), and agarwood (*Aquilaria malaccensis*) were collected from Sylhet region of Bangladesh. Unlike commercial antibiotics, which have side effects on probiotics (beneficiary bacteria), leaf extract activities were analyzed to check if they had positive effects on probiotics that can be found in the gastrointestinal tract as well as dairy products. Potential beneficiary bacteria, *Lysinibacillus macroides* strain SRU-001 (NCBI accession no. MW665108), and pathogenic bacteria, *Aeromonas caviae* strain YPLS-62 (NCBI accession no. MW666783), were isolated from the small intestine of poultry and curd, respectively. Tea and agarwood leaves (5 g powder/80 mL methanol) with solvents were kept for seven days at room temperature, and extracts were applied for antimicrobial assays by the disc diffusion assay against the isolated bacteria. 50 *µ*L of each leaf extract was examined against 50 *µ*L of each bacterial culture, where gentamicin was a control. After 24 hours of incubation, tea and agarwood leaf extracts showed an 11–15 mm zone of inhibition against pathogenic *A. caviae*, while only BT-8 showed 7 mm (disc diameter 6 mm) against probiotic *L. macroides*. However, compared to leaf extracts, gentamicin showed a 27 mm zone of inhibition against both *L. macroides* strain SRU-001 and *A. caviae* strain YPLS-62 bacteria. This research clearly indicates that gentamicin kills both pathogenic and beneficiary bacteria, while leaf extracts from tea and agarwood plants contain antimicrobial activity against only pathogenic *A. caviae* but no effects on probiotic *L. macroides*. This outcome indicates not only the potential therapeutic values of tea and agarwood leaves as antibiotics over commercial antibiotics but also the chance of having pathogens in curd and potential beneficial bacteria from the poultry small intestine.

## 1. Background

Infectious diseases are the most common and sometimes life-threatening illnesses caused by pathogens such as bacteria, viruses, and fungi. Though antibiotics are acknowledged to be life-saving medications, antibiotic resistance is a growing hazard to human health and is difficult or impossible to treat. This is an unfortunate event in that during this same period of time, antibiotic resistance among bacterial strains has increased, thus making it more difficult to treat bacterial infections [1]. Indeed, the mainstream approach in fighting against these diseases is now focused on the modification of existing antibiotics to combat emerging and re-emerging resistance of pathogens globally [2]. We may escape the dire consequences of antimicrobial resistance because there has been a stream of new antibiotics; still, alternative sustainable solutions are required. In previous days, the production of new antibiotics was directly proportional to the development of resistant strains. However, with the help of medical and medicinal technology, a huge improvement has been made in pharmacology and companies are engaging to produce unlimited remedies, while antibiotic resistance still has some challenges. Moreover, the negative impact of commercial antibiotics on both humans and farm animals has recently drawn public awareness and concern. From the ancient time, pharmacological sectors had not been as developed as today, but people had the therapeutic knowledge of traditional medicinal plants and their applications. Plants have several secondary metabolites which are reported to exhibit antibacterial, antifungal, and insecticidal properties [3, 4]. In this research, we selected two types of leaves from tea and agarwood plants to analyze antimicrobial activity. Tea is an infusion of leaves that has been consumed for centuries as a beverage and is valued for its medicinal properties. Due to phenolic compounds, the health beneficial strong antioxidant activity has been found in the tea. The composition of tea phytochemicals can be altered by environmental factors such as soil type, sun exposure, rainfall, or agronomic factors such as culture in greenhouses or fields, biological culture, hydroponic culture, and fruit yield per tree. [5]. According to the process, there are three types of tea: unfermented tea (green and white tea), partially fermented tea (red and oolong tea), and fermented tea (black tea) [6]. Alkaloids, flavonoids, steroids, phenols, and terpenoids are familiar valuable bioactive compounds for medicine development present in green tea leaves [7]. Flavonoids are natural polyphenols present actively in green tea and are commonly known as catechins responsible for antioxidant activities such as free radical neutralization that is developed in the metabolism process [8]; daily moderate consumption of green tea kills *Staphylococcus aureus*, *Vibrio parahaemolyticus*, *Clostridium perfringens*, *Bacillus cereus*, *Plesiomonas shigelloides*, etc. [9]. In the case of black tea, leaves and buds are fermented or oxidized after they have been dried as phytochemicals present in tea leaves which are highly sensitive to the oxidation process. Agarwood (*Aquilaria malaccensis*) is famous in the perfume industry and for other additives, and this plant is widely distributed in south and southeast Asia such as Bangladesh, Bhutan, India, Indonesia, Iran, Malaysia, Myanmar, the Philippines, Singapore, and Thailand. A study on the secondary metabolites of agarwood leaves revealed that leaves contain alkaloids, flavonoids, triterpenoids, steroids, and saponins [10]; these compounds together with saponins and alkaloids have a verified record as antimicrobial agents in many other plants [11]. The use of agarwood is not only limited to resins or perfumes but has also been extended in the pharmaceutical sector with several bioactivities, in particular antimicrobial, antioxidant, analgesic, antiplasmodic, etc. [12, 13]. These plant-based phytochemicals have potential activity against pathogens; however, not all bacteria or microbes are pathogenic, and some are beneficial too. The term “Probiotics” is derived from the Greek words pro (favor) and bios (life). Probiotics are defined as live microbes which are mostly found in the gastrointestinal tract as nonpathogenic bacteria that have a beneficial health effect on adequate quantity consumption in their host as well as can be advantageous for their host by improving the microbial balance in the gut tract [14]. The microbial ecology in the gastrointestinal tract influences many functions such as digestion, absorption of nutrients, detoxification, and ultimately the functioning of the immune system in our body [15, 16]. The intestine's microbial equilibrium is crucially maintained by these microbes and the host's overall health; the bacterial concentration is usually 103 colony forming units/ml, and the most commonly isolated species are *Streptococci*, *Staphylococci*, *Lactobacilli*, and various fungi [17]. The probiotic organism has the property known as “generally recognized as safe” (GRAS) benefits including lactose digestion in lactose-intolerant people, prevention of colon cancer, lowering cholesterol, lowering blood pressure, improving immune functions, vitamin production, and preventing infections [18]. They can also be used in the form of supplements as an alternative to antibiotics [19]. The genus *Lysinibacillus* produces a broad range of antimicrobial bacteriocins against food-borne bacterial and fungal pathogens, isolated from fruits and vegetable waste [20, 21]. The identity of the isolate was established using the colonial description and morphological, biochemical, and molecular characteristics, and sequence analysis revealed the strains in some studies that also isolate bacteria from insects that reside in poultry to be *L*. *macroides*, *Paenalcaligenes hermetiae*, *Bordetella flabilis*, *Bacillus aerophilus*, and *Klebsiella variicola* which showed antibacterial activity against both Gram-positive and Gram-negative clinical isolates [22]. Fermented dairy products are known to be rich in probiotics, and some studies suggested that curd is a good source of *Lactobacillus* bacteria that contain probiotic properties [23]. Probiotic microorganisms are also found in many food products and contain probiotics; for example, fermented food curd and living body probiotics are found both in human and farm animals, especially in their gastrointestinal tract. Addition of probiotics in foods is rising demand in order to enhance its nutritional values [24]. Probiotics are an important supplement in the dairy industry employed in a number of dairy products including sour/fermented milk, yogurt, cheese, butter/cream, ice cream, and infant formula [25]. Prebiotics such as yogurt, cheese, and curd are readily available popular dairy food products enriched with not only probiotics but also supplemented with nutrients compared to other market packaged probiotics. However, the main challenge is using probiotics in maintaining the effective dose of live bacteria at various temperatures in the final product and during the storage period [26]. Curd is one of the most popular fermented milk products and has highly beneficial effects on human health; a great number of lactic acid bacteria (LAB) were found from various fermented foods [27]. Curd is a fermentation of milk made by mixing with lemon or an inoculum of previously made curd and is used in most local market curd production in Bangladesh, popularly known as natural, cheap, and energetic dairy food compared to yogurt and cheese. Despite an ideal source of nutrition with probiotics, curd is an attractive medium for bacterial growth too. Unhygienic food processing increases the possibility of pathogenic attraction in particular, and *Aeromonas* strains have been found in different types of food, such as meat, fish, seafood, vegetables, and processed foods. Potentially, they could represent a serious problem in food, as many strains are able to grow at temperatures of a common refrigerator and at a pH of 4–10, and in the presence of increased salt concentrations, they may pose a major threat to food safety [28]; septicemia, which is typically seen in immune-compromised patients and is triggered by the organism spreading from the intestinal tract to the systems of circulation, is one of the possible causes of extraintestinal infections and respiratory tract infections, such as epiglottitis and pneumonia, as well as less common conditions such as meningitis, peritonitis, ocular infections, and hemolytic uremic syndrome [29]. The water-borne bacteria belonging to the *Aeromonas* genus are widespread; they have been found in sediments, chlorinated water, distribution systems, drinking water, and residual waters, especially during hot months in increased numbers [29]. Mesophilic Aeromonas is becoming a significant pathogen in humans and is responsible for a number of extraintestinal, systemic, and gastrointestinal illnesses. 85% of the clinical isolates of the genus Aeromonas consist of two species and a single biotype of a third species: *A. hydrophila*, *A. caviae*, and *A. veronii* sv. sobria [30]. Novel exciting bacteria *L. louembei* isolated from a traditional fermented food of Congo-Brazzaville was also postulated to produce bacteriocins-like molecules, and biochemical and genotypic characteristics have been tested for the ability to kill pathogenic bacteria such as *Salmonella*, *Staphylococcus*, and *Shigella*; this means that this bacterium could also be a candidate for probiotic features [31]. Antibiotics can affect both types (pathogenic or beneficial) of bacteria, and this study was conducted aiming to compare tea and agarwood leaves and commercial antibiotic effectiveness against bacteria where gentamicin was used as a control.

## 2. Methods

### 2.1. Sample Collection

Tea (*Camellia sinensis*) and agarwood (*Aquilaria malaccensis*) plant leaves were collected from several locations in the Sylhet region in Bangladesh. Three types of tea were collected: fresh leaves (from three varieties BT-6, BT-7, and BT-8); nonfermented tea (green tea); fully fermented tea (black tea); and agarwood fresh leaves. For bacteria, curd and bacterial samples from poultry intestine samples were collected from the local market of Baluchar, Sylhet, and the Department of Biochemistry, Sylhet Agricultural University, Sylhet, Bangladesh, respectively. For the antimicrobial activity test, gentamicin was chosen as the control.

### 2.2. Processing and Extraction of Phytochemicals

Leaves were cleaned and dried in a hot dryer (Biobased) at 45°C. Dried samples were ground by using a blending machine and finally labeled in a separate plastic box. Extraction and processing of six plant leaf (BT-6, BT-7, BT-8, GT, BT, and agarwood leaves) material were carried out. Samples were slightly sterilized and heat-dried not more than 40°C continuously for fine grinding without any damage to phytochemicals. Five grams of powdered samples was weighed and mixed with 80 ml of methanol in a conical flask, covered with aluminum foil. Then, it was kept for 7 days at room temperature for quick extraction of phytochemicals. Whatman filter paper was used for the extraction of the filtrate from the residues and collected slowly in a conical flask and then stored inside an autoclaved Petri dish at 8°C in the refrigerator. The filtrate obtained after filtering each plant extract with Whatman filter paper 1 and the residue obtained were stored at 4°C for further use.

### 2.3. Isolation of Bacteria

The collected local market curd was stored and prepared for sample serial dilution. The minimum number of samples were conducted for serial dilution (1:10) with double-distilled water and cultured in the nutrient agar medium for 24 hours at room temperature. The most dominant single colony was isolated from the agar medium and repeated to isolate the pure colony. The collected bacterial colony-containing agar plates from the poultry intestine were subcultured again and again to obtain the most dominant bacterium. After repeated subculture, both most dominant and pure bacterial isolates from the curd and poultry intestine were stored at −40°C for further analysis. 1 mL bacterial samples were cultured in 9 mL nutrient broth media and kept at 37°C for 24 h. Based on the colonial morphology (i.e., color, size, margin, and shape) of bacteria on agar plates, several bacterial isolates were preserved for further subculturing. The subculturing of isolates was performed in agar media by incubating at 37°C for 24–48 h for expecting *Lactobacillus* as this bacterium is mostly found as probiotics. Isolated colonies were stored at −40°C for further analysis. Further identification and characterization based on different morphological and biochemical tests were examined.

### 2.4. Morphological and Biochemical Characterization

At first, both the samples were inoculated in the nutrient broth medium for enrichment of bacterial growth for 24 h at 37°C aerobically. Pure colonies were cultured on nutrient agar for further morphological, biochemical, and molecular analysis as well as antibiotic susceptibility tests. Morphological tests such as Gram staining and biochemical tests, the citrate test, and the oxidase test were performed [32].

### 2.5. Molecular Identification: 16S rRNA Gene Sequencing and BLAST

Molecular identification was carried out as described in [33]; Promega Maxwell 16 extraction platform DNA was extracted from two samples using the automated Maxwell 16 Cell Total RNA Purification Kit with the Maxwell 16 Instrument (Promega). From total DNA, the 16S rRNA gene was amplified using universal primers 27F and 1492R. The specific PCR for amplification of bacterial 16S rRNA gene fragments was performed by using Hot Start Green Master Mix (dNTPs, Buffer, MgCl_2_, Taq Pol; Cat: M7432, Origin: Promega, USA). The purified DNA reaction mixture was loaded onto a single capillary ABI 3100 DNA analyzer, and chromatograms of the 16S rRNA raw sequence files were read using Chromas software. The 16S rRNA sequences from two isolates were constructed for consensus sequence using Bio-Edit 7. The most closely related strains were identified through sequence comparisons against the NCBI (National Center for Biotechnology Information) GenBank database (https://blast.ncbi.nlm.nih.gov/Blast.cgi) using BLAST (Basic Local Alignment Search Tool) [34]. The 16S rRNA gene sequences of the bacterial isolates were deposited in GenBank under accession numbers MW666783 and MW665108.

### 2.6. Antimicrobial Assays

Antimicrobial activity tests were performed using the disc diffusion method (also known as Kirby–Bauer test methods) [35]. From the subculture microorganisms, the top of each colony was touched with a loop and the colony was transferred into 5-6 mL of nutrient broth. The broth culture was then incubated at 37°C for 24 h to attain the preferable turbidity. Each plate was poured with 15 mL of the Mueller–Hinton Agar (MHA) medium. Then, 50 *µ*L of the bacterial culture was taken using a micropipette and poured onto the plate. Sterile cotton was used for streaking the dried surface of the MHA plate. Blank discs were prepared by using a filter paper. Using sterilized forceps, dried and sterilized blank discs were treated separately with extract soaking in DMSO. The prepared discs containing leaf extracts were air-dried in an aseptic environment. After preparation, the discs were pressed with sterile forceps into the center of an agar plate and the plates were turned over and incubated for 15 minutes at 37°C. Each plate was tested after 24 hours of incubation. The diameter of the discs (6 mm) and their entire zone of inhibition (as determined by naked eyes) were measured, and the zones were measured using a ruler to the nearest whole millimeter where gentamicin (30 *µ*L) was used as the control.

## 3. Results

### 3.1. Isolation of Bacteria

**Figure 1:**
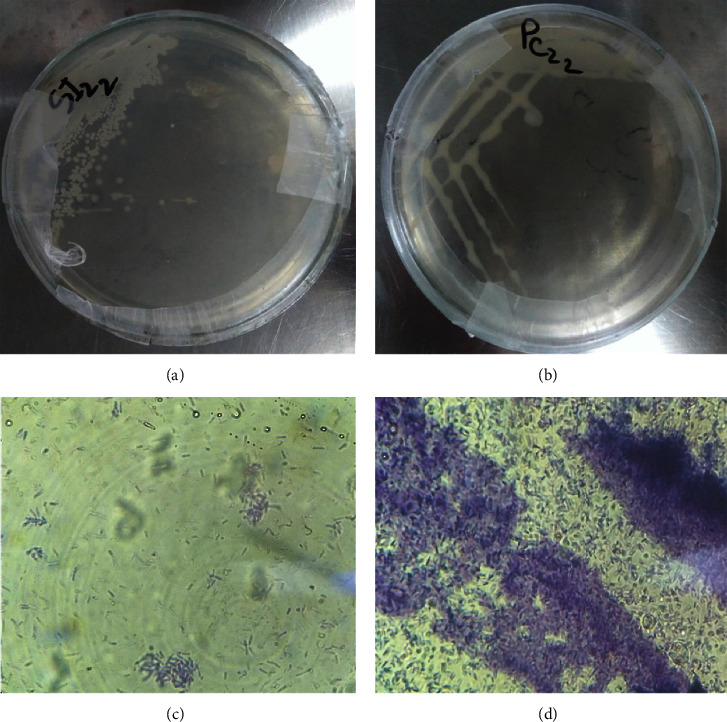
Isolation of bacteria from poultry and curd morphological analysis of (a) isolates from the small intestine (SI) of poultry in the nutrient agar medium and (b) isolates from curd (PC) products in the nutrient agar medium. (c) Isolates from curd under microscopic examination and (d) isolates from the poultry intestine under microscopic examination after Gram staining.

**Figure 2:**
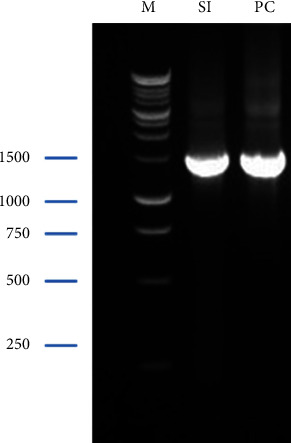
Agarose gel electrophoresis of PC (isolates from curd) and SI (isolates from the poultry intestine) (27F and 1492R primers); M denotes 1 kb DNA ladder.

Two dominant bacteria were isolated from curd and poultry samples. Based on morphological, biochemical, and finally molecular analysis, the bacteria were identified (Table 1; Figures 1 and 2). According to [36] *Lysinibacillus macroides* and [37] *Aeromonas caviae*, morphological and a few biochemical tests were observed.

### 3.2. Molecular Identification

The bacterial species was identified by conducting a nucleotide BLAST analysis on bacteria isolated from a curd sample. The selection criteria for matching the bacteria were a query coverage of more than 97%, a minimum identity percentage of 97.3%, and a maximum identity percentage of 99.5%. BLASTn for the 16s rRNA partial sequence of PC revealed that PC belongs to the class of Gammaproteobacteria and the family of Aeromonadaceae. Finally, it was identified as *Aeromonas caviae* strain YPLS-62 (NCBI accession no. MW666783) and was found closest to the *Aeromonas caviae* strain. Molecularly, it was identified as *Aeromonas* (NCBI accession no. MW666783) and was found closest to *Aeromonas caviae*. Similarly, isolated bacteria from the small intestine were identified by conducting a nucleotide BLAST analysis where selected prerequisites were query cover of more than 97%, the minimum percentage of identity 97.6%, and the maximum percentage of identity 99.7%. BLASTn for the 16s rRNA partial sequence of SI revealed that SI belongs to the class of Bacilli and the family of Bacillaceae. Finally, it was identified as *Lysinibacillus macroides* strain SRU-001 (NCBI accession no. MW665108) and was found to be closest to *Lysinibacillus macroides*.

### 3.3. Comparison of Antimicrobial Activity of Leaf Extracts and Antibiotics against the Bacteria

**Figure 3:**
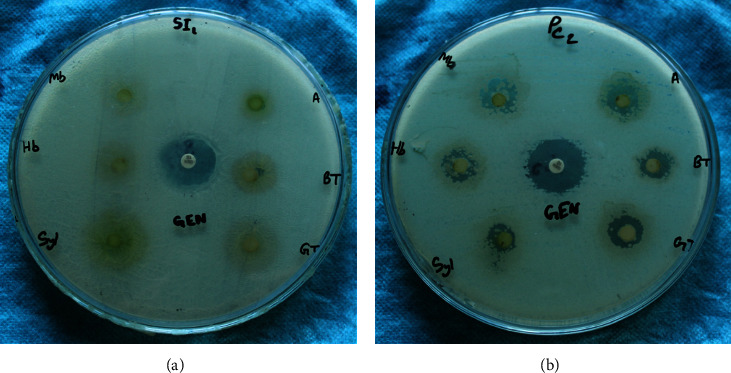
Comparative antimicrobial activity between leaf extract and antibiotic gentamicin (GEN). Leaf extracts Mb = BT-7, Hb = BT-8, Syl = BT-6, BT = black tea, GT = green tea, and *A* = agarwood leaf and antibiotics against (a) SI = isolates *Lysinibacillus macroides* strain SRU-001 from the poultry small intestine and (b) PC = isolates *Aeromonas caviae* strain YPLS-62 from curd products.

All leaf extracts were subjected to two isolates one from curd *Aeromonas caviae* strain YPLS-62 which is pathogenic and another from poultry intestine *Lysinibacillus macroides* strain SRU-001 which is health beneficiary bacteria where gentamicin was used as the control (Figure 3). No zone of inhibition was observed against *L. macroides* strain SRU-001, while almost all leaf extracts showed antimicrobial activity against *A. caviae* strain YPLS-62 (Table 2).

## 4. Discussion

It is urgent to take a step not only in fighting against new and emerging pathogen resistance but also in looking for alternative solutions in a shake of minimizing both the crisis of health and economic loss. However, not all bacteria and fungi are pathogenic; some beneficiary bacteria also have a great impact on human or other physiology; that is why, the trend of adding probiotics to traditional food is not new but demanding mostly. Because of their great health significance, researchers are also trying to find out their medicinal, antimicrobial, and nutritional properties and how to add and store probiotics in popular food. According to one report, the inclusion of probiotics in potato chips can enhance their health benefits, indicating that the addition of probiotics is becoming popular in the food industry [24]. In addition, findings also showed that in order to control *Streptococcus agalactiae* infection and to lower fish mortality, *Enterococcus faecium* ABRIINW.N7 was applied as a probiotic diet for tilapia fish by using herbal gums such as 0.2% Persian gum and 0.4% Fk in combination and 0.8% alginate as encapsulation capable of promoting the growth of probiotic cells in the food environment and digestive tract [38]. In this study, to compare between commercial antibiotics and herbal antibiotics, two types of bacteria were isolated from two different sources, namely, poultry and curd. After bacterial characterization and molecular sequencing, two bacteria were identified: one is *L. macroides* strain SRU-001 (NCBI accession no. MW665108) known as beneficial bacteria from the small intestine of poultry, and another is *A*. *caviae* strain YPLS-62 (NCBI accession no. MW666783) from curd which is pathogenic. After molecular characterization and sequence analysis, researchers also have found *L*. *macroides* which resides in insects of poultry [22]. On the other hand, it is not surprising that often dairy products can be contaminated easily and attract pathogenic bacteria when poor sterility is maintained in the product process and storage. As dairy products are an ideal medium for pathogenic growth in their favorable temperature and environment, there is a chance of contamination by pathogens. Several species of *Aeromonas* are considered emerging pathogens because they cause a wide spectrum of diseases in humans, mainly gastroenteritis, wound infections, and bacteremia/septicemia, infecting immune-compromised and immune-competent patients [29]. However, several researchers have identified that *Lysinibacillus* bacteria have promising characteristics of probiotics such as antimicrobial activity [20, 21]. Researchers have compared the antimicrobial activity between the secondary metabolite from *L*. *macroides* with the standard antibiotic (amoxicillin) activity against *Bacillus subtilis*, *Salmonella*, and *Staphylococcus aureus* [22]. The extract of *L*. *macroides* showed a minimum inhibitory concentration of 60% for *Bacillus subtilis* and 80% for *Staphylococcus aureus* and *Salmonella typhi* [22]. Nevertheless, comparing the antibacterial activity of leaf extracts and synthetic antibiotics, both pathogenic bacteria and probiotics were applied where gentamicin was used as the control. Gentamicin is a widely used third generation antibiotic which is an important part of the medical practice for a long time. Although there is an increasing antimicrobial resistance rate, gentamicin is still a powerful option for many bacterial infections. *Escherichia coli*, *Klebsiella pneumoniae*, *Enterobacter* spp., *Pseudomonas aeruginosa*, and some strains of *Neisseria*, *Moraxella*, coagulase-negative *staphylococci*, and methicillin-susceptible *Staphylococcus aureus* isolates show inhibition against gentamicin in clinical drug concentration, although they can readily develop resistance [39–41]. All leaf extracts were subjected to two isolates: one from curd, that is, *A. caviae* strain YPLS-62 which is pathogenic and another from the poultry intestine, that is, *L. macroides* strain SRU-001 which is health beneficiary bacteria where gentamicin was used as the control. No zone of inhibition was observed against *L. macroides*, while almost all leaf extracts showed antimicrobial activity against *A. caviae*. Similar research has found that silymarin plant extracts and a putative probiotic *Lactobacillus* extract have antifungal activity against *Aspergillus flavus* even when combinations of both plant and *Lactobacillus* extracts have higher antifungal activity than the individual effect [23]. Fresh tea BT-8 showed the highest zone of inhibition (15 mm) against the pathogenic *A*. *caviae* bacteria, and the zone of inhibition of gentamicin was 27 mm against both of the bacteria. This result indicates that tea and agarwood leaf extracts have more antimicrobial activity against pathogenic bacteria than beneficiary bacteria, while commercial antibiotics have the same action against both of these bacteria, and this means that leaf extracts have antimicrobial activity against pathogenic bacteria without diminishing probiotics or health beneficial bacteria. Gentamicin showed an inhibition zone of 27.0 ± SEM mm against both of the bacteria, while leaf extracts showed negligible inhibition (6/7 mm where disc diameter is 6 mm) against probiotic *L*. *macroides* strain SRU-001 but showed more than 10 mm zone of inhibition against pathogenic *A*. *caviae* strain YPLS-62. Besides containing health beneficial bioactive compounds, these leaves can help people get rid of pathogenic infection and gain sound health by consuming them as a drink. The results suggest that tea and agarwood leaves have a close effect on antimicrobial performance against pathogens. However, this study's research limitations can be extended or improved by the application of a broad range of commercial antibiotics and pathogenic bacteria, probiotics, market probiotics, biochemical screening of the isolated bacteria, and minimum inhibitory concentrations as well as novel antimicrobial compound isolation in the future. From the above discussion, it can be concluded that tea and agarwood leaves can play the role of potential antibiotics against pathogens and reduce the detrimental effect of antibiotic resistance. One of the side effects of synthetic antibiotics is reducing essential microbial flora which can be overcome by the mentioned plants also. Therefore, natural herbal therapy after commercial production can withstand antibiotic resistance without causing harm to intestinal essential microbial flora. Considering all the factors and parameters, it may be concluded that production of natural antibiotics from tea and agarwood leaves has the potential to fight against antibiotic resistance and boost up immunity.

## 5. Conclusion

This study suggests tea and agarwood leaves as alternatives or additives to synthetic antibiotics. Tea is consumed as a popular beverage worldwide, and the health benefits of both tea and agarwood are widely recognized. The present study concluded that the two plant leaves under investigation exhibit antibacterial activity, thereby highlighting one of their beneficial effects. The inhibition activity of these leaf extracts and synthetic or commercial antibiotics was also analyzed against isolated beneficial bacteria and other pathogenic bacteria, whereas the side effects of synthetic antibiotics, for instance, reducing probiotics, were also observed, and tea and agarwood leaf extracts showed a potential alternative solution. Antimicrobial activity of leaf extracts recorded significant positive results than synthetic antibiotics against pathogenic bacteria and beneficial bacteria. Curd is an excellent source of probiotics, but unhygienic handling could be a chance for pathogenic growth, and common people are sometimes unaware about this as well as consuming commercial antibiotics that not only kill pathogenic but also kill beneficiary bacteria. One of the beneficiary effects of probiotics is antimicrobial activity, and in this case, *Lysinibacillus macroides* strain SRU-001 can play a role; in addition, tea and agarwood plant leaves may act as a blessing in the case of saving beneficiary bacteria though more advanced research is required for standardization of phytochemicals as natural antibiotics. However, to identify the core mechanism of the bacterial defense system as well as documentation of leaf extracts, phytochemical determination and antimicrobial bioactive compound analysis and determination of minimum inhibitory concentration by the microdilution method can be investigated to explore novel antimicrobial compounds in the future for better understanding.

## Figures and Tables

**Table 1:** Morphological and biochemical characterization of isolated bacteria.

Name of the isolated sample	Sources	Morphological properties	Biochemical properties
*Lysinibacillus macroides*strain SRU-001 (SI)	Poultry	Gram-positive, diploid in size, irregular round shape, motile	MRS test: positive
			Oxidase test: negative
			Catalase test: positive
			Citrate test: positive

*Aeromonas caviae* strain YPLS-62 (PC)	Curd	Gram-negative, creamy, irregular rod shape	MRS test: poor growth
			Oxidase test: positive
			Catalase test: negative
			Citrate test: positive

**Table 2:** Leaf extracts exhibit antibacterial activity against probiotic bacteria *Lysinibacillus macroides* strain SRU-001 and other pathogenic bacteria *Aeromonas caviae* strain YPLS-62.

Bacteria	Name of leaf extracts						Control gentamicin
	Zone of inhibition (mean ± SEM)						
	GT	BT	BT-8	BT-7	BT-6	Agarwood	
*Lysinibacillus macroides* strain SRU-001	6.0 ± 0.0	6.3 ± 0.5	7.0 ± 1.7	6.0 ± 0.0	6.6 ± 1.1	6.0 ± 2.3	27.0 ± 1.0
*Aeromonas caviae* strain YPLS-62	14.3 ± 4.0	14.0 ± 3.6	15.0 ± 2	12.0 ± 3.6	11.0 ± 2.6	11.0 ± 1.7	27.0 ± 1.0

## Data Availability

The data used to support the findings of this study are available from the corresponding author upon request.
